# Job Satisfaction and Optimal Experience in a Swedish Governmental Administration—A Mixed Methods Study

**DOI:** 10.3390/bs15060720

**Published:** 2025-05-23

**Authors:** Fredrik Molin

**Affiliations:** 1 IPF, The Institute for Leadership and Organizational Development, Uppsala University, Kungsängsgatan 5B, 753 22 Uppsala, Sweden; fredrik.molin@ipf.se; 2 Department of Medical Sciences, Occupational and Environmental Medicine, Uppsala University, 752 37 Uppsala, Sweden

**Keywords:** job satisfaction, optimal experience, public organization, mixed methods, work engagement, work conditions

## Abstract

Background: Job satisfaction and optimal experiences, often linked to employee engagement and performance, are critical factors for organizational performance. This study investigated job satisfaction and the conditions enabling optimal experiences among employees within a Swedish governmental administration. This study sought to fill a critical gap in understanding job satisfaction within a governmental setting. Method: A mixed methods approach was utilized to collect both quantitative and qualitative data, combining survey data (*n* = 117) and workshop interviews (*n* = 14). The quantitative component included measures of job satisfaction, learning, and optimal experience. Results: Optimal experience was strongly associated with job satisfaction and the positive use of knowledge and skills at work. The results also indicated high levels of job satisfaction among participants and frequent opportunities for optimal experience in daily work. The qualitative findings revealed that while employees often felt supported in their roles, organizational constraints and limited autonomy could hinder the consistent occurrence of optimal experience. Participants emphasized the importance of clear communication, prioritization, and a supportive work environment. Conclusion: This study contributed to the understanding of workplace dynamics in governmental organizations, emphasizing the interplay between job satisfaction, knowledge and skills, and optimal experience. The findings underscore the need for addressing organizational barriers to optimal employee experiences within a bureaucratic setting.

## 1. Introduction

The concept of flow has emerged as an aspect of understanding optimal human experiences in various professional settings. Coined by psychologist Mihaly Csikszentmihalyi, flow refers to a state of deep absorption, focus, and intrinsic motivation that individuals experience when fully engaged in an activity (Csikszentmihalyi, 1975, 1990, 2014). Job satisfaction and flow represent two interconnected constructs that play roles in understanding the dynamics of employee experiences within the workplace. Job satisfaction, a widely studied phenomenon, refers to the positive emotional and attitudinal responses individuals have towards their work (Locke, 1969). The interplay between job satisfaction and optimal experience has implications for the overall well-being and productivity of employees (Nierenberg et al., 2017; Slemp & Vella-Brodrick, 2014).

Knowledge workers, often engaged in intellectually demanding tasks, face a distinctive set of challenges and opportunities that influence their ability to attain and sustain flow states (Alvesson, 2001, 2004; Davenport, 2005). A knowledge worker is defined by (De Sordi et al., 2021) as “professionals whose work is highlighted by the continuous, systematic and predominant expansion of organizational knowledge through the mechanism of exploration”(De Sordi et al., 2021, p. 65). Investigating optimal experience and how it manifests in this specific professional context holds implications for the well-being and job satisfaction of employees but also for organizational productivity and effectiveness (Drucker, 1999; Eisenberger et al., 2005).

### 1.1. The Theory of Flow

Csikszentmihalyi’s Flow Model identifies three key conditions that need to be in place to be able to experience flow. These are as follows: (1) clear goals and objectives for the activity; (2) immediate feedback, also referred to as micro-feedback, during the activity; and (3) a balance between challenge and skill (Csikszentmihalyi, 1990, 2014). When these elements align, individuals are more likely to enter a state of flow, resulting in heightened concentration, enjoyment, and a sense of fulfillment (Csikszentmihalyi, 1990). Flow is experienced when there is a perfect balance between the task’s difficulty and one’s skill level at a high proficiency. It is in a state of flow that creativity and innovation thrive. This experience is closely tied to mental well-being, as individuals who are frequently in flow tend to exhibit greater emotional stability and subjective well-being (Nakamura & Csikszentmihalyi, 2002).

Flow is closely linked to intrinsic motivation, where individuals engage in activities for the inherent satisfaction and enjoyment rather than external rewards (Deci et al., 1999; Deci & Ryan, 2000; Ryan & Deci, 1985). The autonomy and mastery aspects of intrinsic motivation are particularly relevant to the flow experience, as individuals seek challenges that align with their skills and derive satisfaction from the process itself.

Experiencing flow, the state of being fully immersed and engaged in an activity, is more common at work than during leisure (Bryce & Haworth, 2002; Csikszentmihalyi & Lefevre, 1989). This counterintuitive finding can be attributed to the clear frameworks, structures, and goals that typically characterize work environments. These elements provide individuals with explicit guidance and a sense of purpose, creating conditions conducive to flow. In contrast, leisure activities often lack such inherent structure, requiring individuals to independently select and organize activities, which can make it harder to achieve the deep focus and challenge–skill balance necessary for flow. Flow at work is also associated with tasks that are perceived as meaningful and challenging, especially when employees have the autonomy to approach their work creatively (Demerouti et al., 2012). Structured feedback and the presence of achievable, clearly defined goals further enhance the likelihood of experiencing flow at work. In leisure, however, the absence of external motivators and frameworks can lead to a lack of direction, making it less likely for individuals to achieve a state of flow unless they actively create structure themselves. Thus, the contrast between work and leisure in fostering flow highlights the importance of aligning tasks with personal skills and goals within a structured context.

### 1.2. Flow in Different Contexts

A growing body of research has examined the phenomenon of flow across professional, educational, and cultural domains, applying a range of methodological approaches. In a comprehensive meta-analysis, Liu et al. (2023) highlighted enjoyment and intrinsic motivation as central predictors of work-related flow. Notably, enjoyment emerged as the strongest predictor of job performance, while intrinsic motivation was more closely tied to life satisfaction. Dai and Wang (2025) investigated language teachers in China and found that those with proactive personalities experienced greater flow and work engagement—pointing to the value of cultivating proactive dispositions in academic environments. Similarly, Chen et al. (2025) focused on the connection between flow and teachers’ innovative behavior, identifying core antecedents such as challenge–skill balance, clear goals, and immediate feedback as being crucial in fostering creativity. Sun (2025) explored auditory influences, demonstrating that background music can enhance flow among undergraduates, while Tse et al. (2022) analyzed flow across age groups and concluded that age itself was not a significant factor, despite notable individual variability. From a neurocognitive standpoint, Gold and Ciorciari (2020) proposed that targeted interventions could enhance flow potential. In the fitness domain, Oh and Kim (2023) found that smartwatch features support flow during exercise by improving goal clarity and feedback. These studies point towards a growing understanding of the factors that influence flow across various settings and populations. The evidence suggests that flow is a multifaceted phenomenon, influenced by individual traits such as personality and motivation, as well as external conditions like environmental cues and task-specific factors.

### 1.3. Job Satisfaction Theories

Herzberg’s classic two-factor theory (Herzberg, 1966) proposes that job satisfaction, job dissatisfaction, and motivation are influenced by distinct factors. Hygiene factors, such as working conditions and salary, can prevent dissatisfaction but may not necessarily contribute to satisfaction. Motivational factors, including recognition and achievement, are crucial for enhancing job satisfaction. Locke (1969), on the other hand, argued that job satisfaction is predominantly a positive emotional state resulting from the appraisal of one’s job or job experiences. Job satisfaction is multi-dimensional, i.e., it is influenced by factors such as work conditions, pay and benefits, recognition and achievement, and autonomy. The Job Characteristics Model (JCM) by Hackman and Oldham (1975) posits that certain job characteristics contribute to job satisfaction. Key elements include skill variety, task identity, task significance, autonomy, and feedback. Jobs that incorporate these characteristics are more likely to foster positive attitudes and increased job satisfaction among employees. Providing people with control over their work serves to improve mental health, job satisfaction, and performance (e.g., the job characteristics model (Hackman & Lawler, 1971)).

Contemporary theory on job satisfaction defines job satisfaction as the extent to which individuals like or dislike their jobs, encompassing both overall feelings and specific facets such as pay, supervision, and the nature of the work (Spector, 2022). Job satisfaction is linked to important outcomes for both employees and organizations. Compared to the dissatisfied, satisfied employees have better mental and physical health, higher work engagement, are more likely to remain in the organization, and are more productive (Judge et al., 2020; Spector, 2022). Salary and monetary remuneration may contribute to job satisfaction by addressing basic needs and providing financial security, which are foundational elements of well-being (Judge et al., 2010). However, research suggests that beyond a certain threshold, the impact of salary on job satisfaction diminishes, and non-monetary factors such as meaningful work, workplace relationships, and opportunities for personal growth play a more significant role (Chadi & Hetschko, 2018; Hoff et al., 2020). Some scholars emphasize that true satisfaction often stems from intrinsic rewards, such as a sense of purpose and alignment with personal values, rather than solely from extrinsic incentives like pay (Ryan & Deci, 1985; Savery, 1989). Consequently, achieving a balance between financial compensation and fulfilling job characteristics is critical for fostering a sustainable and deeply satisfying work environment (Barling et al., 2003; Judge et al., 2010; Watson & Slack, 1993).

### 1.4. The Relationship Between Flow and Job Satisfaction

The relationship between job satisfaction and flow is bidirectional. Jobs that provide opportunities for flow are likely to contribute to higher job satisfaction, as individuals find intrinsic fulfillment in their tasks. Conversely, satisfied employees are more likely to experience flow due to their positive attitudes and engagement with their work. The interconnectedness of these constructs indicates the importance for organizations seeking to enhance both employee satisfaction and performance (Eisenberger et al., 2005). Theoretically, it is possible for flow to occur independently of satisfaction; for example, an individual may find a task engaging yet misaligned with their personal goals or values, which could hinder overall satisfaction (Seligman, 2011). Conversely, one may achieve satisfaction from an outcome—such as receiving a favorable grade or successfully completing a project—even in the absence of flow, particularly in cases where the process involved considerable effort or was unenjoyable (Ryan & Deci, 2000). Such dynamics suggest an asymmetrical relationship; while flow can contribute to satisfaction, it is neither a necessary nor sufficient condition for its occurrence. Moreover, this relationship may be moderated by various factors, including individual differences (such as need for cognition or intrinsic motivation) (Ryan & Deci, 1985), the nature of the task, or the contextual environment (differentiating between leisure activities and work settings). For instance, the satisfaction derived from experiencing flow during leisure pursuits may function differently compared to flow experienced in high-pressure work situations, where external demands can alter the perceived meaning and ramifications of the experience (Csikszentmihalyi, 1997).

Research on optimal experiences within government settings remains limited. In a qualitative interview study, Jones (2021) identified five key elements that public service employees associate with positive work experiences: challenge, efficacy, camaraderie, empowerment, and service. Among these, challenge emerged as the most reported factor, suggesting that employees thrive in demanding and engaging roles. These results indicate that creating conditions for optimal experiences at work is possible in the public sector, and emphasizing these characteristics could improve job satisfaction among public sector employees.

In the workplace, flow has been associated with increased job performance, creativity, and overall job satisfaction (Burke, 2010). The demands of knowledge workers, often requiring cognitive engagement and problem-solving (De Sordi et al., 2021), create an environment conducive to flow experiences. While the phenomenon of flow has been extensively studied in diverse contexts, there remains a gap in the literature concerning its manifestation within the white-collar workforce, particularly in the setting of governmental administrations. The Swedish governmental administration chosen for this study serves as a microcosm, reflecting the broader trends and patterns prevalent among educated and skilled public sector officials. By investigating the white-collar job satisfaction and optimal experience in this setting, this study aims to contribute insights that may inform workplace policies, organizational structures, and individual strategies to enhance employee well-being and performance. This study thus aims to investigate job satisfaction and the conditions for optimal experience among a sample of Swedish Transport Administration employees.

## 2. Materials and Methods

### 2.1. Design

A mixed methods study design was selected to meet the contextual demands of the research, which required both quantitative and qualitative perspectives to address the complexity of the topic. Specifically, a convergent mixed methods design was employed, as described by Creswell and Clark (2017). This design facilitates the simultaneous collection and analysis of quantitative and qualitative data, enabling their integration to produce a comprehensive understanding of the research problem. The choice of this approach was informed by the advantages of mixed methods design in capturing unexpected findings through the combination of diverse data sources, as highlighted by (Bryman & Bell, 2011).

### 2.2. Participants

This study involved employees from various departments within the Swedish Transport Administration. The Swedish Transport Administration is a government agency responsible for the long-term planning of the transport system and has approx. 10,000 employees. A total of 14 workshops were conducted, encompassing diverse functions such as HR, Finance, Communication, Law, and Planning, as well as business areas (Major Projects, Maintenance, Planning, and Traffic Control), and a profit unit. The goal was to capture job satisfaction and optimal experience across different white-collar roles within the Administration.

### 2.3. Data Collection

From fall 2016 to spring 2017, 14 workshops with a total of 128 participants were organized within the Administration. Each workshop lasted two hours. On average, eight participants attended each workshop, with the number of attendees ranging from five to eleven. The selection criteria for the workshops were crafted to ensure a diverse representation across various sectors within the Administration. To fulfill this criterion, participant recruitment was carried out in collaboration with the Administration. Data collection was deemed complete once saturation was achieved concerning organizational diversity. With one exception, each workshop comprised participants exclusively from a single unit within the organization; in the second workshop, however, participants from both the Economy and Planning functions were included.

Participants were invited through an internal contact at the Administration and the invitation read as follows: “Invitation to workshop. As part of the research project ‘Management and motivation’, you are hereby invited to a research workshop where we discuss how individuals’ driving forces affect the conditions for creativity and innovation in daily work. The workshop provides an opportunity to talk with colleagues about the conditions of everyday work and about the details of the work that you don’t always have time to reflect on, but which can still be decisive for the work to function. As a participant, you will make important contributions to the research project ‘Management and motivation’ and, by extension, to the development of the Swedish Transport Administration’s internal processes and working methods”.

Informed consent was obtained following the introduction of the workshop. Participants were informed that their involvement was voluntary and that they could withdraw at any time without any negative consequences. Additionally, they were assured of the complete anonymity and confidentiality of their data, with no personal identifiers being included in the final analysis or reporting. Ample opportunity was provided for participants to ask questions about how their data would be used, stored, and safeguarded, ensuring they fully understood the scope and purpose of the research. All procedures adhered to the principles outlined in the Helsinki Declaration, emphasizing ethical conduct, respect for participants, and the protection of their rights throughout the study.

The workshops lasted two hours and were structured as follows:

1. Introduction: Providing an overview of the workshop and informed consent for participation in the study.
2. Survey: Administering a questionnaire covering background data, optimal experience, and inquiries related to learning and job satisfaction.
3. Background Theory: Offering participants a theoretical framework to understand the concept of flow and its application in their work contexts.
4. Small Group Work: Participants engaged in discussions and activities aimed at mapping flow conditions and experiences based on their individual work situations.
5. Large Group Summary: A collaborative session where participants shared insights, discussed commonalities, and summarized the key findings from the small group exercises.

During small group discussion sessions, participants reflected on and described their work situations based on the three conditions for flow: (1) clear goals and objectives for the activity; (2) immediate feedback, also referred to as micro-feedback, during the activity; and (3) a balance between challenge and skill. The discussions were captured through group summaries and visual representations on blackboards and flipcharts. In addition, a research assistant documented the plenum summary discussions led by the author using a word processor.

Participants in the workshop were asked to fill out a questionnaire containing four question areas (background information, optimal experience, questions about knowledge and skills, and job satisfaction). The questionnaire was distributed at the beginning of the workshops after a short introduction by the author. The questionnaire consisted of 20 items. See Table 1 for the sample items.

To reduce the time spent on the questionnaire, six items were chosen from the Dispositional Flow Scale (DFS-2) (S. Jackson et al., 2008), measured on a five-point Likert scale. The items were selected to represent the key dimensions of the flow construct, such as concentration, control, and enjoyment, ensuring the scale remained both concise and representative. Participants rated their agreement with each statement, ranging from 1 (“strongly disagree”) to 5 (“strongly agree”). The decision to use a reduced set of six items was made with the goal of balancing construct validity with practical considerations of survey length and participant burden. The six selected items correspond to the core dimensions of the flow experience identified by Csikszentmihalyi (1990, 1997), namely balance between skill and challenge, clear goals, unambiguous feedback, concentration on task, sense of control, and autotelic experience. These dimensions are considered central antecedents and experiential components of flow across a wide range of domains (S. A. Jackson & Eklund, 2002). They also align closely with the original phenomenological model of flow, making them particularly suitable for parsimonious yet valid measurement.

For knowledge and skills, four items selected from the QPSNordic questionnaire (Dallner et al., 2000) were used. These items were chosen to measure the key aspects of knowledge, required skills, and active participation in the workplace environment.

For job satisfaction, a single-item question was utilized to capture participants’ overall perception of their satisfaction with their job (Wanous et al., 1997). This approach provided a straightforward measure, allowing respondents to quickly express their general feelings about their work experience. Previous studies have shown that single-item measures of job satisfaction correlate highly with multi-item scales. Wanous et al. (1997) found, in a meta-analysis, a mean correlation of r = 0.63 between single-item and multiple-item satisfaction scales, suggesting that global measures are psychometrically robust. An advantage of single-item measures is that they reduce participant burden, especially in studies where space is limited, and this increases response rates and reduces fatigue without significantly compromising validity (Matthews et al., 2022). Allen et al. (2022) argued for the adoption of single-item measures by highlighting their main benefits: they are time-efficient, they can be less ambiguous than multi-item measures, and they are often more satisfying for respondents. These advantages make single-item measures particularly suitable when looking for a straightforward assessment of a specific concept, especially in situations requiring quick data collection, or in situations where participants’ time is limited. For these reasons, a single-item measure of job satisfaction was employed in the present study.

### 2.4. Data Analysis

Survey data were analyzed using descriptive statistics, frequency distributions, and cross-tabulations. The qualitative data obtained from the workshops were thematically analyzed to provide an interpretation of the flow experiences within the Swedish Transport Administration. Results from discussions in the workshops were analyzed using qualitative content analysis (Hsieh & Shannon, 2005). This method is characterized by systematically categorizing and breaking down textual data to answer the research questions (Graneheim & Lundman, 2004).

The analysis was conducted in several steps: The first step was a first read-through of the complete workshop material. In the second step, the material was read through once again—now with the main research questions in focus. In the third step, the material was coded first openly (i.e., inductively) and then with the specific aspects of flow in mind, as well as the hampering and supporting factors for flow identified in the material. This coding process unfolded in multiple stages. The first round of coding, following the initial acquaintance with the material, involved what Miles et al. (2014) referred to as descriptive coding or open coding. In this stage, the content was described using codes that linked directly to the corresponding sections of the text, also referred to as data-driven content analysis (Forman & Damschroder, 2008). The primary objective of this initial round was to summarize data segments into more manageable units. In vivo coding was employed (Miles et al., 2014), which involved selecting codes that closely mirrored the actual content and utilized terminology presented in the text passage. Subsequent coding focused on identifying patterns and recurring themes within the data.

The data were coded by the author and subsequently discussed with a fellow researcher to ensure comprehensive and rigorous analysis. This collaborative approach served multiple purposes: it facilitated the validation of the coding process, provided an opportunity for critical feedback, and allowed for the identification of any potential biases in interpretation. The author presented the initial coding framework, along with the rationale behind the chosen codes, to the colleague, who then offered insights and perspectives that may not have been previously considered. This collaborative effort aimed to ensure the rigor and trustworthiness of the findings (Shenton, 2004), ensuring that the interpretations drawn from the data were both robust and reflective of the underlying phenomena.

## 3. Results

### 3.1. Quantitative Results

A total of 117 participants completed the flow survey (91.4%). Background data indicated that 38% of respondents were male, and 62% were female. The mean age was 48.0 years (SD 9.33), with the oldest respondent being 64 years old and the youngest 25 years old. A total of 14.5% of the respondents indicated that they had a managerial position. The average length of employment was 12.3 years (SD 10.30), ranging from less than a year to 42 years. Notably, 22% of respondents had worked for more than 20 years within the Swedish Transport Administration.

Concerning future employment, 9.4% of respondents did not plan to continue working in the Swedish Transport Administration in the longer term (3–5 years). With half of them being older than 61, this suggested they had planned retirement.

Regarding learning at work, 11.1% of respondents indicated they learned new things every day, with none reporting never learning new things. Regarding job satisfaction, the average response on a five-point scale was 4.25 (SD 0.73) and 41% of the respondents responded with a five on the scale, indicating high job satisfaction. The results are presented in Table 2.

Cronbach’s alpha for the knowledge and skills scale was 0.62, which fell below the recommended threshold of 0.70 (DeVellis, 2003). This suggests that while the items showed some variability, they still correlated meaningfully enough to be considered acceptable for preliminary analysis—particularly given that alpha is sensitive to the number of items in a scale. The average inter-item correlation was 0.28, which fell within the recommended range of 0.20 to 0.40. This indicated that the items were sufficiently homogeneous to reflect a common construct yet retained enough unique variance to avoid redundancy. In contrast, Cronbach’s alpha for the optimal experience scale was 0.74, indicating acceptable internal consistency.

Cross-tabulations showed no significant differences between male and female participants regarding job satisfaction, knowledge and skills, and optimal experience (Wilcoxon rank sum test, *p* = 0.202 for job satisfaction).

Correlations between constructs are shown in Table 3, indicating a significant positive correlation between optimal experience and job satisfaction (r = 0.548) and between learning and job satisfaction (r = 0.625).

The assumption of normality for the optimal experience and knowledge and skills variables was assessed using both visual and statistical methods. Visual inspection of the histograms indicated that the distributions for both constructs were approximately normal, with no evident skew or kurtotic deviations. This impression was supported by analyses of skewness and kurtosis. Optimal experience showed a skewness value of −0.378, and knowledge and skills had a skewness of −0.302. These values fell within the accepted range of −1 to +1, indicating a relatively symmetric distribution. Additionally, kurtosis values were within acceptable bounds, suggesting no significant issues with peakedness or flatness. Together, these results support the assumption of normality for both variables, justifying the use of parametric statistical analyses in subsequent steps.

A multiple regression analysis was conducted to examine the relationship between job satisfaction and the proposed predictors. In this analysis, job satisfaction was the dependent variable, while learning and optimal experience were the independent variables. The findings indicated that both learning and optimal experience had a positive impact on job satisfaction, with learning exerting a stronger influence. Both predictors were statistically significant (*p* < 0.001). The regression model was significant and accounted for 48% of the variance in job satisfaction (Adj. R^2^ = 0.4808), suggesting that nearly half of the variation in job satisfaction can be explained by the combined effects of learning and optimal experience. The results are summarized in Table 4.

To assess the potential for multicollinearity among the constructs, the Variance Inflation Factor (VIF) was calculated. In this analysis, the VIF = 1.213, which was below the accepted thresholds of 5 (moderate concern) or 10 (serious concern), indicating that multicollinearity was not a concern for the variables. The relatively low VIF confirmed that the constructs contributed unique information to the model, supporting the validity of the regression analyses.

The control variables sex and age were included in the multiple regression model to account for potential demographic influences on the outcome variable. However, neither variable emerged as a significant predictor. Specifically, age showed a non-significant effect (B = −0.004, SE = 0.007, *t* = −0.56, *p* = 0.576), indicating that differences in age did not significantly relate to the outcome. Similarly, sex (coded as 1 = female, 2 = male) also had no significant effect on the dependent variable (B = −0.175, SE = 0.138, *t* = −1.27, *p* = 0.208). These findings suggest that gender and age did not contribute meaningfully to the variance in the outcome variable within this model.

### 3.2. Workshop Results

The results of the workshops conducted related to the three primary conditions for flow: clear goals, challenge and skill level, and micro-feedback, along with additional themes that emerged during the discussions.

A predominant theme that emerged from the workshop discussions was the necessity of having clear goals. Participants emphasized that ambiguity in task objectives leads to confusion and a lack of motivation. Participants noted that the organization’s overarching vision and values often felt disconnected from their daily tasks. One participant expressed, “It is difficult to apply the Administration’s vision and values to daily work. There is a gap between the visions and the goals you set for yourself” (WS 9—Communication). This disconnect not only impeded the ability to set clear personal goals but also diminished the overall sense of purpose in the work being performed. Moreover, the discussions highlighted the need for a structured framework that outlines priorities and the rationale behind them. As one participant noted, “There is an ambiguity in what is important; it is difficult to know how important things are” (WS 8—Maintenance). This lack of clarity contributed to a culture where everything appeared equally important, leading to stress and reduced motivation, according to the participants.

A recurring theme throughout the discussions was the uncertainty surrounding task priorities. Participants articulated that ambiguity regarding what is deemed important can lead to a significant loss of energy and creativity. The culture of the organization, which often prioritized accessibility and responsiveness, was seen as a barrier to maintaining focus and achieving flow. One participant poignantly remarked, “Our organization functions on the principle that all tasks are seen as equally significant, which makes prioritization unfeasible” (WS 1—Economy). This reflected a broader issue within the organization, where the lack of clear priorities could lead to confusion and disengagement among employees. Participants suggested that improving the organization’s mandate and providing transparent reasons for task prioritization could help mitigate these challenges.

Participants reported that the challenge and skill level associated with their tasks significantly influenced their ability to experience flow. Many acknowledged possessing the relevant knowledge and skills necessary for their work, yet faced challenges stemming from task complexity, urgency, and high quality requirements. The pressure of time constraints was particularly noted as being a factor that heightened the difficulty of tasks, often leading to stress rather than a productive flow state. The complexity of tasks, coupled with conflicting interests and the need to balance various priorities, emerged as significant barriers. Participants expressed that the demanding nature of their work often left little room for creative thinking and engagement. One participant summarized this sentiment by stating, “The expectation of availability and the priority of incoming matters impede attention and concentration” (WS 9—Communication). In contrast, participants identified project-based work as a more conducive environment for achieving flow. Projects typically come with well-defined goals and time constraints, allowing individuals to concentrate better and engage meaningfully with their work. According to the respondents, the structured nature of projects aligns with flow conditions, fostering an environment where employees can immerse themselves in their tasks.

The concept of micro-feedback was another critical area of discussion among participants. While some individuals reported that they received feedback during their tasks, many expressed that this feedback was often insufficient or unclear. The lack of clear micro-feedback signals was attributed to both the nature of the activities and a deficiency in participant training to recognize existing feedback cues. As noted by several participants, establishing effective signals for self-monitoring is essential for fostering flow. Participants expressed a desire for more structured feedback mechanisms that could help them gauge their performance and make necessary adjustments in real-time.

The discussions revealed that concentration and attention were critical components of the flow experience. Participants reported facing numerous distractions in their work environment, which hindered their ability to focus. The culture of accessibility, characterized by an expectation to be constantly available, was identified as a significant impediment to maintaining concentration. Several participants highlighted the challenges posed by open office landscapes, which, while intended to promote collaboration, often resulted in increased distractions. As one participant noted, “The work environment should be flexible enough for me to choose a setting that aligns with my approach to tackling the task at hand” (WS 6—Major Projects).

Participants expressed challenges associated with governing documents, which often lacked clarity and were perceived as overly controlling. The multitude of bureaucratic guidelines and policies inhibited employees’ freedom in executing their tasks, leading to frustration and confusion. As one participant noted, “While there are many policies and guidelines that provide considerable flexibility in implementation, it’s important to streamline them into fewer documents” (WS 6—Major Projects). This sentiment reflected a broader desire for simplification in the overall organizational management control. Participants emphasized that providing reasons for task priorities and clarifying the purpose of governing documents could enhance employees’ understanding and engagement.

The workshops highlighted the importance of awareness regarding flow conditions in designing activities and structuring work. Participants expressed that the increased awareness gained from the workshops allowed them to reflect on their work structures and task distributions more critically. This awareness is essential for creating an environment that nurtures creativity and engagement. However, the prevailing organizational culture, which prized constant accessibility and multitasking, often conflicted with the individual’s need for uninterrupted focus. One participant articulated this tension, stating, “When I close my office door, it feels like I am doing something wrong” (WS 9—Communication).

## 4. Discussion

The aim of the study was to investigate job satisfaction and the conditions for optimal experience among a sample of Swedish Transport Administration employees. The quantitative results show a high level of job satisfaction among the respondents and that optimal experience is clearly linked to both job satisfaction and learning. The participants state that they can experience flow during their work. At the same time, the qualitative results indicate that this experience is hindered by organizational constraints.

The results from the study indicate a variability in how employees perceive and experience flow. This suggests that individual differences play a significant role. Factors such as personal motivation, work style, and the nature of specific tasks contribute to the subjective experience of flow (Csikszentmihalyi, 1990; Nakamura & Csikszentmihalyi, 2002). This underscores the importance of recognizing and understanding individual circumstances and preferences.

The qualitative findings point to the design of the office as a source of frustration among employees. Open office designs have become increasingly popular in modern workplace environments. This shift away from traditional cubicles and private offices reflects a broader trend toward fostering collaboration and a sense of community within organizations (Oldham & Brass, 1979). However, it is important to recognize that the benefits of open office arrangements are not universally experienced (Gerlitz & Hülsbeck, 2024). While this is not reflected in the quantitative results of the current study, it may be argued that the impact of such designs can vary significantly depending on factors such as the nature of the work, employee personality traits, and the prevailing organizational culture (Kim & De Dear, 2013; Wohlers & Hertel, 2018). For example, although some individuals may flourish in environments that promote easy interaction, others may find that the lack of privacy and heightened noise levels negatively affect their concentration and overall productivity (Bernstein & Turban, 2018). This suggests that open office designs, while potentially beneficial for some, may not be suitable for all work settings or employee preferences (Bakker et al., 2014). Additionally, the potential for distractions and interruptions in an open office setting might lead to heightened stress levels and decreased job satisfaction for those who prefer more quiet and solitary workspaces (Seddigh et al., 2015). Therefore, though the quantitative data may not capture these nuances, qualitative insights from the workshops point to how open office designs may affect morale, productivity, and overall workplace dynamics. In addition, the qualitative results also indicate a work culture that emphasizes emergent tasks over planned tasks. Such ambiguity regarding what is deemed important can lead to significant energy loss and decreased creativity (Mintzberg, 1994; Nielsen & Cleal, 2010).

Psychological models such as flow—while useful for understanding individual motivation and engagement—can be limited in public organizational contexts due to structural and institutional constraints. The emphasis on autonomy, feedback, and a balance between challenge and skill may be constrained in public bureaucracies, which often operate within rigid governance structures, strict procedural frameworks, and hierarchical decision-making processes (c.f. Lipsky, 1980/2010). This can make the conditions necessary for achieving flow difficult to sustain. Additionally, institutional priorities in public organizations—such as accountability, prudence, and conformance to regulations—may conflict with the dynamic and creative conditions that psychological models often assume. As a result, applying such models without considering these broader organizational and governance factors can lead to unrealistic expectations. While psychological models offer valuable insights, their application in public sector settings must be contextualized within the constraints of institutional structures and bureaucratic norms.

### 4.1. Avenues for Future Research

Future research should address several key areas to deepen the understanding of flow and its relationship with performance. Harris et al. (2023) suggested that future studies should further explore qualitative insights and the nuances of the flow experience. They identified potential biases in the existing literature and highlighted the value of incorporating qualitative perspectives to enrich findings. Similarly, Dai and Wang (2025) called for the inclusion of diverse methodologies, such as interviews and mixed methods approaches, to better capture the dynamic interplay of flow components across different contexts and cultures. Although the present study utilized a mixed methods design, future research could benefit from incorporating more qualitative data, such as interviews, conducted in various contexts, to further validate and expand upon these results.

Another area for future research is the realm of bureaucratic politics and public administration. The results from the present study point to several organizational constraints that may be specific to politically controlled bureaucracies. Investigating how political oversight, policy cycles, and administrative cultures shape day-to-day work experiences could offer insights into how flow and job satisfaction manifest in public sector contexts. Moreover, integrating theories from bureaucratic politics, such as principal–agent dynamics or street-level bureaucracy, could deepen our understanding of how structural and political factors interact with individual-level psychological states.

### 4.2. Implications for Practice

The findings highlight the need to simplify the governance structures and clarify the purpose of governing documents. By consolidating policies and providing clear reasons for task priorities, employees’ understanding and engagement can be enhanced. Leadership emerges as a factor in fostering an environment conducive to optimal experiences and mitigating the above-mentioned barriers (Bass & Riggio, 2006).

Based on the findings of this study, some recommendations, in order of priority, can be made to enhance optimal experiences among employees within a governmental setting: (1) Establish clear priorities and processes: Revisit governing documents to streamline processes and clarify roles and responsibilities. Promote a culture that values planned work and provides time for deep engagement (Newport, 2016). (2) Promote open communication: Create forums for dialog where employees can voice concerns and suggestions regarding workload management and task prioritization (Edmondson, 1999; Stöllman et al., 2025). (3) Train leaders on flow and concentration: Provide training for leaders to recognize and facilitate conditions for flow, emphasizing the importance of minimizing disruptions and supporting employees in their work (Grant, 2012). (4) Engage employees in office design decisions: Conduct surveys and focus groups to gather employee feedback on office design and its impact on their work experiences. Consider implementing design changes that accommodate diverse working styles (Hattie & Timperley, 2007; Wohlers & Hertel, 2018).

The implication of the results is that these findings can guide policy and organizational changes to enhance employee job satisfaction and retention in a Swedish governmental administration. By identifying factors that promote optimal experiences, the research supports initiatives aimed at improving work culture and employee engagement. This can lead to higher productivity, better public service delivery, and healthier work environments, ultimately fostering a more effective and satisfied workforce in the public sector.

### 4.3. Limitations

This study has several limitations that should be considered when interpreting the results. Firstly, it is based on a single case study conducted within a governmental administration, specifically the Swedish Transport Administration. While this provides valuable insights into the experiences of white-collar workers within this context, the findings may not be generalizable to other sectors or organizational settings. Even though this study is concentrated on Sweden, the themes and findings resonate with other governmental bodies, particularly in similar Nordic or European contexts. However, it is acknowledged that generalizing the results to other groups should be approached with caution. Secondly, the reliance on self-reported experiences of flow introduces potential biases. Participants’ subjective accounts may be influenced by individual perceptions, recall inaccuracies, or social desirability, which could affect the validity of the data. Furthermore, this study does not account for objective measures of flow, such as performance metrics or physiological indicators, which might provide a more comprehensive understanding. Addressing these limitations in future research through comparative studies across different organizations and the incorporation of mixed method approaches could enhance the reliability and applicability of the findings.

Another limitation of the present study is the use of single-item measures to assess job satisfaction. While such a measure offers the advantage of being quick and cost-effective, it may lack the depth and nuance provided by multi-item scales. As a result, a single-item measure may not capture the full complexity of job satisfaction. Although the measure provides immediate insights, it should be interpreted cautiously. While a single-item measure can be valuable for broad data collection, it may not fully capture the intricacies of job satisfaction, limiting the depth of analysis in this study. Future research could address this limitation by incorporating more detailed measurement tools to enhance the richness of the findings.

One limitation of this study lies in the internal consistency of the knowledge and skills scale, which yields a Cronbach’s alpha of 0.62. However, it is important to note that alpha is sensitive to the number of items in a scale. Given that this is a short, four-item scale, lower alpha values are not uncommon and do not necessarily indicate poor reliability (Cortina, 1993). Nevertheless, the modest alpha suggests that the scale’s internal consistency is only moderate, and as such, results derived from this scale should be interpreted with caution. This limitation may affect the precision of measurement and, potentially, the robustness of the conclusions drawn from this construct. Future research may consider revising or expanding the scale to improve its psychometric properties.

### 4.4. Conclusions

This study of optimal experiences among employees of the Swedish Transport Administration reveals insights into the factors that facilitate or hinder flow within a white-collar setting. The results reveal a high level of job satisfaction and an optimal experience among white-collar workers within the Administration and that the optimal experience is linked to both job satisfaction and learning. Participants in the study reported that they frequently experience flow during their work, which contributes positively to their overall job satisfaction and professional development. However, despite these positive findings, participants also identified several organizational constraints that hinder their ability to experience flow consistently. These constraints include bureaucratic documents, unclear communication, and limited autonomy in the prioritization of tasks. Such factors can disrupt focus, reduce engagement, and undermine the potential for employees to fully immerse themselves in their work. The findings highlight the dual nature of workplace experiences: while employees perceive many opportunities for engagement and satisfaction, structural issues remain that, if resolved, could further optimize their experiences and promote a culture of continuous learning and development.

## Figures and Tables

**Table 1:** Measures and sample items used in this study.

Construct	Sample Item	Previous Source
Optimal experience [6 items]	“I was totally absorbed by what I did”.	Adopted from the DSF-2 scale (S. Jackson et al., 2008)
Knowledge and skills [4 items]	“My work provides positive challenges”.	QPSNordic (Dallner et al., 2000)
Job satisfaction [1 item]	“I am satisfied with my work”.	(Wanous et al., 1997)

**Table 2:** Level of learning, job satisfaction, and optimal experience among participants.

Variable	Score	N	Min	Max	M	SD
Job satisfaction	1–5	117	2	5	4.248	0.730
Knowledge and skills (four items)	4–20	117	10	20	15.735	2.306
Optimal experience (six items)	6–30	117	14	29	22.137	3.124

Note: Cronbach’s alpha for knowledge and skills (0.621) and optimal experience (0.739).

**Table 3:** Correlation among variables.

Variable	1	2	3
1. Job satisfaction	---		
2. Knowledge and skills	0.6246 **	---	
3. Optimal experience	0.5483 **	0.4192 **	---

Note: ** *p* < 0.001 Pearson r.

**Table 4:** Linear regression on job satisfaction.

	b	SE	t	*p*
Constant	−0.25234	0.43319	–0.583	0.561
Learning	0.17173 **	0.02642	6.500	<0.001
Optimal experience	0.08122 **	0.01722	4.716	<0.001

Note: ** *p* < 0.001, N = 117; Adj. R^2^ = 0.4808; F(2, 114) = 54.71, *p* < 0.001.

## Data Availability

Data are contained within the article.
